# Correction: Mitema, A. et al. The Development of a qPCR Assay to Measure *Aspergillus flavus* Biomass in Maize and the Use of a Biocontrol Strategy to Limit Aflatoxin Production. *Toxins* 2019, *11*, 179

**DOI:** 10.3390/toxins11070384

**Published:** 2019-07-01

**Authors:** Alfred Mitema, Sheila Okoth, Suhail M. Rafudeen

**Affiliations:** 1 Plant Stress Laboratory 204/207, Department of Molecular and Cell Biology, MCB Building, Upper Campus, University of Cape Town, Private bag X3, Rondebosch, Cape Town 7701, South Africa; 2 Department of Botany, School of Biological Sciences, University of Nairobi, P.O. Box 30197, Nairobi 00100, Kenya

The authors wish to make the following correction to their paper [1]:

In the Introduction section, the fourth sentence of the second paragraph should be replaced with, “Additionally, Mideros et al. [15] developed and validated two RT-qPCR assays to estimate *A flavus* biomass in maize tissues using *Af2*, *Zmt3*, *INCW2-97* and *α-tubulin* marker genes, and studied the relationship between fungal biomass and aflatoxin accumulation.”

In the Results section, the title of the Section 2.1 should be replaced with, “*Colonisation of Plant Tissues by A. flavus*”. The title of Table 1 should be replaced with, “Phenotypic characteristic measurements of control and infected (GAF4 and KDV1) maize lines with Aspergillus flavus isolate KSM014 (*n* = 3) taken after 14 days of growth. Massive variation was observed in roots and shoots of both maize lines, with KDV1 exhibiting more severe symptoms of stunted growth.” The title of the Section 2.3 should be replaced with, “*In-Vitro Biocontrol Strategies in Aflatoxin Management and Aspergillus Flavus*”.

In the Discussion section, the second sentence of the third paragraph should be replaced with, “The current study indicated that the *β*-*Tub* gene was a more suitable marker for the detection of *A. flavus* in maize tissues compared to *Ef1*α and was therefore used in fungal biomass determination.” The second sentence of the ninth paragraph should be replaced with, “It is therefore important to first determine the impact of these environmental factors on aflatoxin production by *A. flavus* KSM012 under laboratory and field conditions to ensure its safe use and efficacy as a biocontrol agent.”

We apologize for any inconvenience caused to readers of Toxins by this change.

The changes do not affect the scientific results. The manuscript will be updated and the original will remain online on the article webpage. We apologize for any inconvenience caused to our readers.
