# Supplementary material for: The Development of a qPCR Assay to Measure Aspergillus flavus Biomass in Maize and the Use of a Biocontrol Strategy to Limit Aflatoxin Production
Source: Toxins (Basel). 2019 Mar 25;11(3):179. doi: 10.3390/toxins11030179 (PMC6468655; doi:10.3390/toxins11030179)
Supplement: Supplementary file 1 [file toxins-11-00179-s001.pdf]

Supplementary Materials: The Development of a qPCR Assay to Measure Aspergillus flavus Biomass in Maize and the Use of a Biocontrol Strategy to Limit Aflatoxin Production

Alfred Mitema, Sheila Okoth and Suhail M. Rafudeen

Table S1. Extracted fungal DNA ($\log_{10}$) from healthy and infected maize tissues (KDVI and GAF maize lines.

Isolate	Control Tissue		KDV1 Maize Line		GAF 4 Maize Line	
			Infected Tissue			
	Aspergillus flavus (ng/µl)	Healthy Plant (ng/µl)	Roots	Shoots (ng/µl)	Roots (ng/µl)	Shoots (ng/µl)
1	0.095	0	1.364877	1.742853	0.8866012	2.109021
2	0.095	0	0.439227	0.6932703	0.2812233	0.4079242
3	0.1498	0	0.721782	0.5111399	0.6285084	-0.2757575
4	0.0258	0	2.036252	1.846066	0.06390438	-1.053601

Table 2. Comparison of extracted fungal DNA concentrations from control and infected maize tissues for GAF4 and KDV1 respectively, according to 1-way ANOVA, Tukey's multiple comparison test and Post linear trend analysis ($p < 0.05$) illustrating significant test variances. (*: variance; sig: significance).

Maize Line	1-Way ANOVA (P < 0.05)	Tukey's Multiple Comparison Test (P < 0.05)	Post-Test Linear Trend (P < 0.05)
	Control vs Infected	Control vs Infected	Control vs Infected
GAF4	No sig. variance ($p < 0.05$)	No sig. variance ($p < 0.05$)	No significant linear trend
KDV1	Sig. variance ($p < 0.05$) **	Sig. variance ($p < 0.05$) *	No significant linear trend
		Control roots vs Infected roots*	
		Control roots vs Infected shoots*	
		Control shoots vs Infected shoots*	
		Infected roots vs Infected shoots	
